# Targeting Src-mediated Tyr216 phosphorylation and activation of GSK-3 in prostate cancer cells inhibit prostate cancer progression *in vitro* and *in vivo*

**DOI:** 10.18632/oncotarget.1770

**Published:** 2014-02-07

**Authors:** Anna Goc, Belal Al-Husein, Katerina Katsanevas, Alison Steinbach, Uvette Lou, Harika Sabbineni, David L. DeRemer, Payaningal R. Somanath

**Affiliations:** ^1^ Clinical and Experimental Therapeutics, College of Pharmacy, University of Georgia, Augusta, GA; ^2^ Charlie Norwood VA Medical Center, Augusta, GA; ^3^ Department of Medicine, Cancer Center and Vascular Biology Center, Georgia Regents University, Augusta, GA

**Keywords:** Src, GSK-3, Tyr-216, prostate cancer, dasatinib

## Abstract

Recent studies suggest a positive correlation between glycogen synthase kinase-3 (GSK-3) activation and tumor growth. Currently, it is unclear how both Akt that inhibits GSK-3 and active GSK-3 are maintained concurrently in tumor cells. We investigated the role of GSK-3 and the existence of an Akt-resistant pathway for GSK-3 activation in prostate cancer cells. Our data show that Src, a non-receptor tyrosine kinase is responsible for ^Y216^GSK-3 phosphorylation leading to its activation even when Akt is active. Experiments involving mouse embryonic fibroblasts lacking cSrc, Yes and Fyn, as well as Src activity modulation in prostate cancer cells with constitutively active (CA-Src) and dominant negative Src (DN-Src) plasmids demonstrated the integral role of Src in ^Y216^GSK-3 phosphorylation and activity modulation. Inhibition of GSK-3 with SB415286 in PC3 cells resulted in impaired motility, proliferation and colony formation. Treatment of PC3 cells with the Src inhibitor dasatinib reduced ^Y216^GSK-3 phosphorylation and inhibited proliferation, invasion and micrometastasis in vitro. Dasatinib treatment of athymic nude mice resulted in impaired growth of PC3 cell tumor xenograft. Together, we provide novel insight into the Src-mediated ^Y216^GSK-3 phosphorylation and activation in prostate cancer cells and reveal the potential benefits of targeting Src-GSK-3 axis using drugs such as dasatinib.

## INTRODUCTION

Prostate cancer is the second leading cause of cancer-related death among men in the United States. Although androgen deprivation therapy is an effective treatment during the early stages of prostate cancer, due to the uncertainty in the molecular mechanisms leading to advanced stages of the disease, patients with castration-resistant prostate cancer are left with limited treatment options which includes chemotherapy, immunotherapy, or novel oral agents such as abiraterone acetate or enzalutamide.

Hyper-activation of the PI3 Kinase-Akt pathway due to PTEN mutation is one of the most common reasons for prostate cancer [1]. An activating E17K mutation in Akt has also been linked to the development of prostate cancer [2], demonstrating that Akt activity is indispensable for prostate cancer development. Our previous studies have demonstrated that changes in Akt activity results in the modulation of prostate cancer cell survival, proliferation, colony formation and tumor growth [3-5] as well as micrometastasis of prostate cancer cells via inside-out activation of the cell surface integrin α_v_β_3_, thus aiding the cellular recognition of specific extracellular matrix (ECM) proteins abundant in the vascular basement membrane and bone [6, 7]. Glycogen synthase kinase 3 (GSK-3), a serine-threonine kinase is one of the least characterized substrate of Akt in prostate cancer cells, whose activity is inhibited by Akt via phosphorylation at serine 9 and 21 in GSK-3α and GSK-3β isoforms, respectively [8, 9]. Since Akt and GSK-3 activities are known to be regulated reciprocally, it is not clear how these two kinases can be simultaneously present in their active form in cancer cells. This demands further investigation on the specific role of GSK-3 in cancer cells and in order to characterize the existence of an Akt-independent mechanism of GSK-3 activity regulation in multiple cancers.

The role of GSK-3 in various cellular functions has been controversial. For example, cell migration, an integral aspect of invasiveness and metastasis of cancer cells is promoted by the local inhibition of GSK-3, but global inhibition impairs cell spreading and migration [10]. Similarly, controversial data were demonstrated when cell proliferation and apoptosis was examined. Some studies have shown that GSK-3β knockout mice are sensitized to apoptosis and die in the embryonic stage [11], while others have shown that over-expression of GSK-3β induces apoptosis [12]. Nevertheless, due to its importance in numerous of cellular functions, GSK-3 activity is tightly regulated via phosphorylation at multiple serine, threonine and tyrosine residues by various kinases. In addition to the Akt phosphorylated Ser21/9 residue, previous studies have indicated the existence of a tyrosine residue in GSK-3 (Y279 in GSK-3α and Y216 in GSK-3β) that may be necessary for its complete activation [13-15]. However, the tyrosine kinase responsible for the phosphorylation of GSK-3 Y216 is still under investigation.

It has been reported that mostly GSK-3β expression is up-regulated in many types of cancer, including prostate cancer [16-21]. Analyses of prostate cancer patient samples have demonstrated a positive correlation between increased levels of cytoplasmic GSK-3β, clinical stage and Gleason score [22]. Compared to normal prostate, GSK-3α and GSK-3β were up-regulated in prostate cancer with GSK-3α elevated in low Gleason tumors and GSK-3β expressed in high Gleason tumors [23]. Furthermore, a study by Liao *et al*. indicated that ^Y216^GSK-3β phosphorylation is elevated in highly aggressive prostate cancer cells [24]. Nonetheless, inhibition of GSK-3 decreases the survival and/or proliferation of many types of cancer, both *in vitro* and *in vivo [25, 26]* demanding additional research in this area.

It has been previously demonstrated that Src family kinases (SFKs), which include cSrc, Yes, Fyn, Lyn and Yes etc. are often de-regulated in multiple cancers, including prostate cancer, and cause aberrant regulation of many cellular processes involved in tumor progression and metastases [27-32]. In the current study, we investigated if Src is involved in the Y216 phosphorylation and GSK-3 activity regulation, and characterized the efficacy of targeting Src-GSK-3 pathway using pharmacological inhibitors for prostate cancer therapy. Here, we report that GSK-3 inhibition decreases cell survival, proliferation, migration, invasion and micrometastasis of prostate cancer cells *in vitro*. We also demonstrate that GSK-3β activation by Src-mediated Y216 phosphorylation augments prostate cancer cell function *in vitro* and tumor xenograft growth *in vivo*. Our study also unveils the potential benefits of dasatinib in the management of prostate cancer by inhibiting Src- GSK-3β pathway, although further pre-clinical and clinical validation is desirable.

## RESULTS

### GSK-3 activity is necessary for prostate cancer cell tumorigenic and metastatic potential

**Figure 1:**
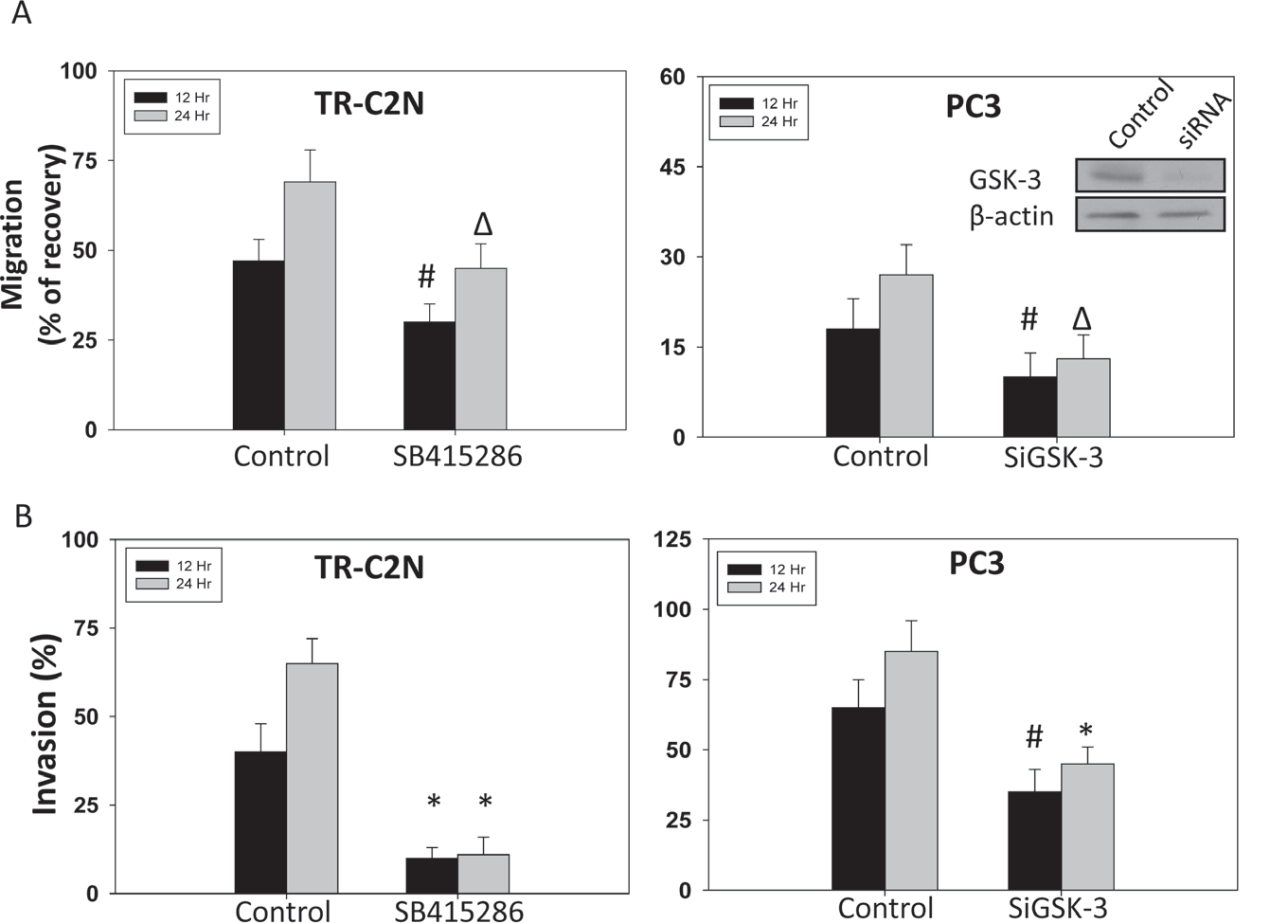
GSK-3 inhibition and gene knockdown impairs migration and invasion of prostate cancer cells Murine metastatic TRAMP (TR-C2N) cells were treated with 50 nM EGF plus 20 μM of GSK-3 inhibitor (SB415286), and human metastatic PC3 cells transiently transfected with SiRNA for GSK-3 followed by treatment with EGF were plated for migration and invasion assays for 12 h and 24 h. Control cells were either treated with 50 nM EGF or transient transfected with scrambled SiRNA. A) Bar graph showing decreased motility of TR-C2N and PC3 cells after GSK-3 activity inhibition and gene knockdown, respectively. B) Bar graph showing decreased invasiveness of TR-C2N and PC3 cells after GSK-3 activity inhibition and gene knockdown, respectively. The data are presented as mean ± SD (n=3) of triplicate experiments (* *p*<0.001, Δ *p*< 0.01, # *p*<0.05 vs. control experiments within the same group).

**Figure 2:**
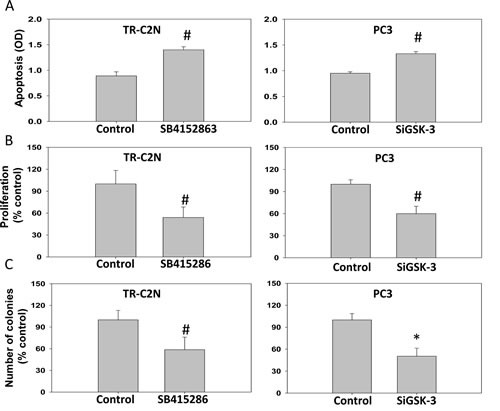
GSK-3 inhibition and gene knockdown impairs cell survival, proliferation and colony formation of prostate cancer cells Murine metastatic TRAMP (TR-C2N) cells were treated with 50 nM EGF plus 20 μM of GSK-3 inhibitor (SB415286), and human metastatic PC3 cells transiently transfected with SiRNA for GSK-3 followed by treatment with EGF were subjected for apoptosis, proliferation and colony formation assays. Control cells were either treated with 50 nM EGF or transient transfected with scrambled SiRNA. A) Bar graph showing increased TR-C2N and PC3 cell apoptosis after GSK-3 activity inhibition and gene knockdown as determined based on cytoplasmic histone-associated DNA fragments detection after 12 h incubation. B) Bar graph showing decreased proliferation rate of TR-C2N and PC3 cells with GSK-3 activity inhibition and gene knockdown as detected by BrDU Labeling and Detection. C) Bar graph showing decreased colony formation by TR-C2N and PC3 cells with GSK-3 activity inhibition and gene knockdown as quantified using by the NIH ImageJ software. The data are presented as mean ± SD (n=4) of triplicate experiments (* *p*<0.001, Δ *p*< 0.01, # *p*<0.05 vs. control experiments within the same group).

Literature on the role of GSK-3 in cancer is controversial. Hence, we first attempted to test the effect of specific inhibition of GSK-3 activity using SB415286 and SiRNA-mediated GSK-3 knockdown in invasive human and murine prostate cancer cells. Since motility and invasion are the hallmarks of invasive and highly metastatic cancer cells we determined whether GSK-3 activity is necessary for these functions in prostate cancer cells. Thus, murine metastatic TRAMP (TR-C2N) cells and human metastatic PC3 cells were examined using pharmacological (SB415286) and genetic (siRNA-GSK-3) approach. Our results indicated that direct inhibition of GSK-3 using inhibitor and SiRNA significantly lowered the rate of cell migration and invasion (Figure 1A and B). Optimal dose of GSK-3 inhibitor was determined by Trypan blue staining based PC3 cell viability assay at 12 h and a cell migration assay at 12 and 24 h, after treatment at 0, 1, 5, 10, 20 and 50 μM doses of SB415286 (Supplemental Figure 1A and B). Moreover, we also observed that inhibition of GSK-3 affects tumorigenic features of prostate cancer cells such as apoptosis, proliferation, and colony formation. Our study revealed that both mouse and human metastatic prostate cancer cell lines exhibited impaired cell survival, proliferation and colony/foci formation (Figure 2A, B, C). Taken together, these results indicated that GSK-3 activity is necessary for prostate cancer cell tumorigenic and invasive properties *in vitro*.

### Modulation of Src activity correlates with corresponding changes in GSK-3 Y216 phosphorylation and its activity

**Figure 3:**
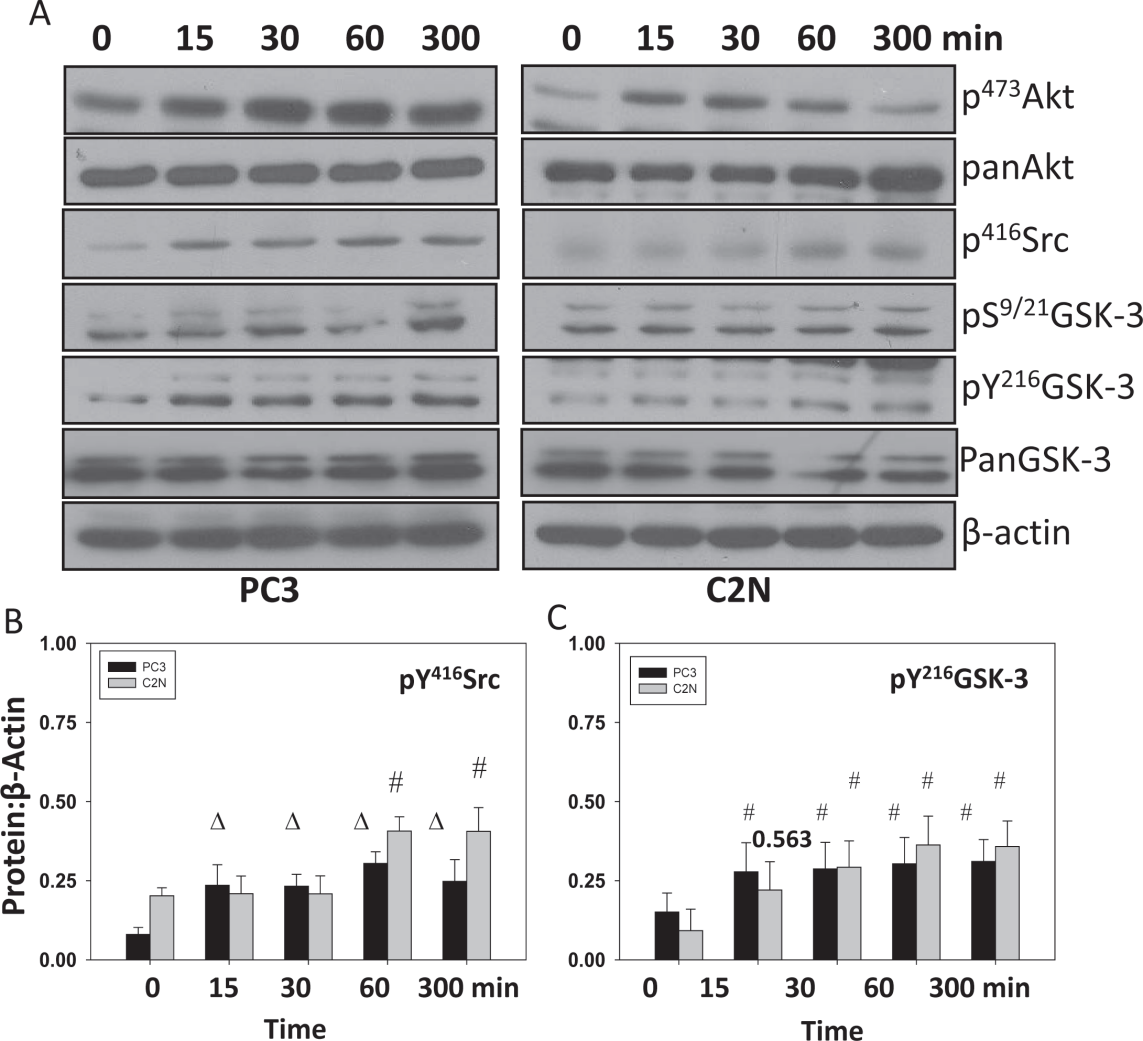
EGF treatment increases phosphorylation of ^Y416^Src and ^Y216^GSK-3 concurrently A) Human metastatic PC3 and murine metastatic TRAMP (TR-C2N) cells were treated with 50 nM EGF and cell lysates were prepared at different time points (0, 15, 30, 60 and 300 minutes). Figure shows Western blot analysis of cell lysates for changes in phosphorylation of ^S473^Akt, ^Ser9/21^GSK-3, ^Y216^GSK-3 and ^Y416^Src, compared to actin. B and C) Bar graph showing densitometry analysis of PC3 and C2N prostate tumor cell lysates as explained above for ^Y416^Src and ^Y216^GSK-3, respectively. The data are presented as mean ± SD (n=4) (Δ *p*< 0.01, # *p*<0.05 vs. control experiments within the same group).

**Figure 4:**
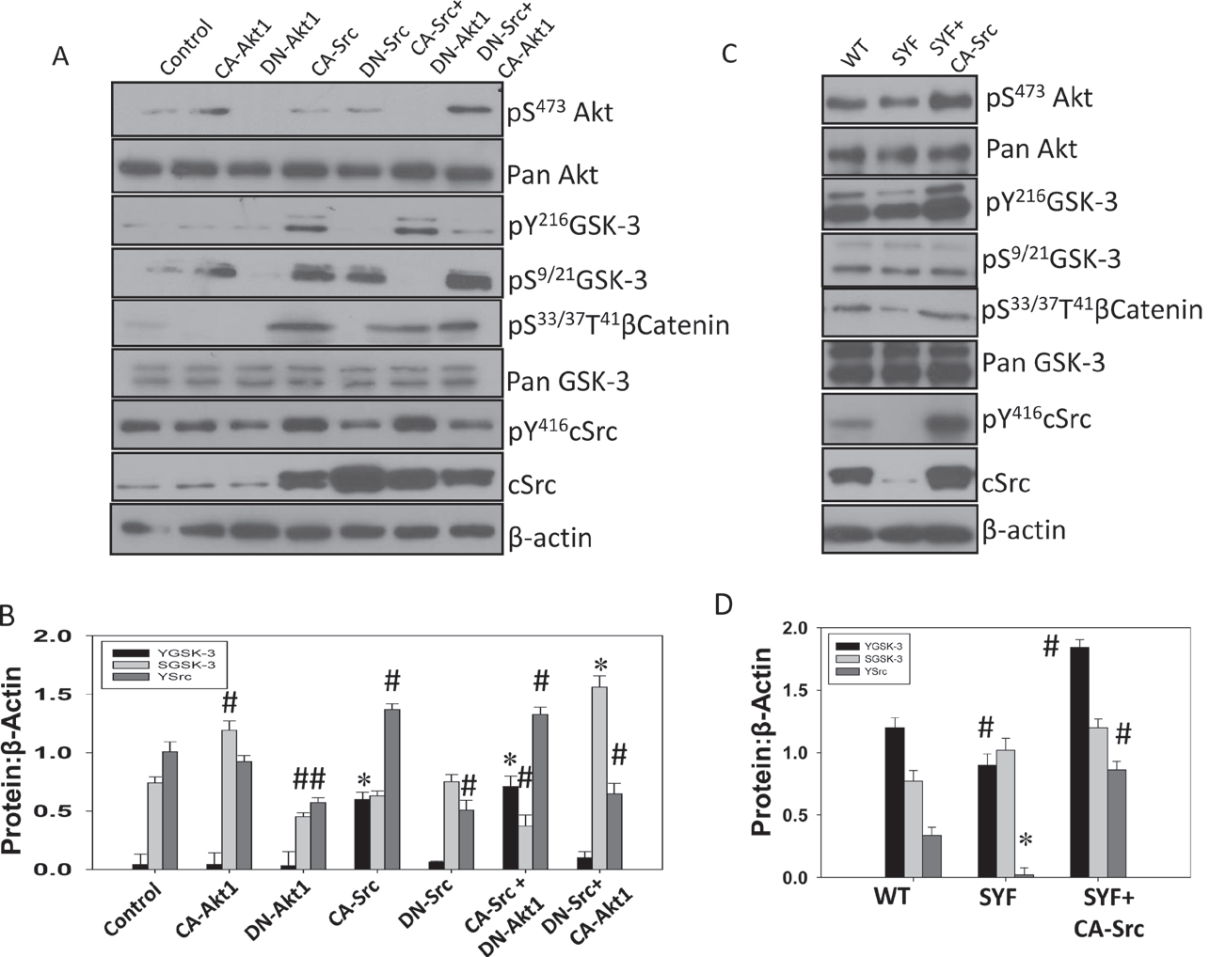
Src activity is necessary for phosphorylation of GSK-3 Y216 in mouse embryonic fibroblasts and PC3 cells A) Transiently transfected human prostate cancer (PC3) cells expressing plasmids for empty pBabe-Puro-vector (control), CA-Akt1, DN-Akt1, CA-Src, and DN-Src, or a combination of CA-Src with DN-Akt1 and DN-Src with CA-Akt1 were lysed and subjected for Western blot analysis for changes in phosphorylation and expression level of Akt, GSK-3, Src and βCatenin. B) Bar graph showing densitometry analysis of PC3 cell lysates as explained above for ^Y416^Src, ^S9/21^GSK-3 and ^Y216^GSK-3, respectively. C) WT and SYF (cSrc, Yes and Fyn triple knockout) mouse embryonic fibroblasts transiently transfected with CA-Src plasmids were lysed and subjected for Western blot analysis for changes in phosphorylation and expression level of Akt, GSK-3 Src and βCatenin. Control cells were transiently transfected with empty pBabe-Puro-vector. D) Bar graph showing densitometry analysis of PC3 cell lysates as explained above for ^Y416^Src, ^S9/21^GSK-3 and ^Y216^GSK-3, respectively. The data are presented as mean ± SD (n=3) (* *p*<0.001, Δ *p*< 0.01, # *p*<0.05 vs. control experiments within the same group).

It has been shown that Akt has the potential to inhibit GSK-3 activity via phosphorylation of its Ser9/21 in α and β isoforms, respectively [33-35]. Although ^Y216^GSK-3 phosphorylation has been linked to its activation [13, 36], the tyrosine kinase responsible for this phosphorylation is still under investigation. Since Src over-expression has been correlated with highly aggressive stages of prostate cancer [37], we sought to determine if Src family kinases (SFKs), cSrc in particular is responsible for Y216 phosphorylation of GSK-3 and its subsequent activation. Our results indicated that treatment of PC3 cells with epidermal growth factor (EGF) significantly increased phosphorylation of ^S473^Akt, ^S21/9^GSK-3, ^Y416^Src and ^Y216^GSK-3 in a time-dependent manner (Figure 3A-C). We then determined the effect of changes in Akt and Src activity in the modulation of GSK-3 phosphorylation in its respective serine and tyrosine residues. Our data indicated that while Akt activity modulation correlated with changes in the Ser9/21 of GSK-3, changes in cSrc activity modulation using specific constitutively active and catalytically inactive mutants correlated with changes in the phosphorylation of ^Y216^GSK-3 and ^Ser33/37/Thr41^βcatenin, a known substrate of GSK-3, thus indicating that Src is responsible for ^Y216^GSK-3 phosphorylation and its activation (Figure 4A and B). While over-expression of PC3 cells with CA-Src increased ^Y216^GSK-3 and ^Ser33/37/Thr41^βcatenin phosphorylation, over-expression with DN-Src resulted in reduced ^Y216^GSK-3 and ^Ser33/37/Thr41^βcatenin phosphorylation. In order to further confirm this result, we utilized mouse embryonic fibroblasts deficient in cSrc, Yes and Fyn isoforms (SYF cells), which has more than 80% of its total SFK phosphorylation reduced. SYF cells indicated a significant, but not total reduction in ^Y216^GSK-3 and ^Ser33/37/Thr41^βcatenin phosphorylation (Figure 4C and D). In contrast, reconstituting the SYF cells with CA-Src resulted in a significant increase in ^Y216^GSK-3 and ^Ser33/37/Thr41^βcatenin phosphorylation, once again indicating that Src is responsible for the tyr216 phosphorylation of GSK-3. Interestingly, significant decrease in phosphorylation level of ^Y416^Src was noticed with over-expression of DN-Akt but contrasting effect with over-expression of CA-Akt1 was not observed. However, over-expression of DN-Src did not affect phosphorylation status of neither ^S473^Akt nor ^S9/21^GSK-3 as presented in Figure 4A and B. Together our results indicated that Src activity is necessary for the Y216 phosphorylation of GSK-3.

### Pharmacological inhibitor of SFKs reduces ^Y216^GSK-3 phosphorylation

**Figure 5:**
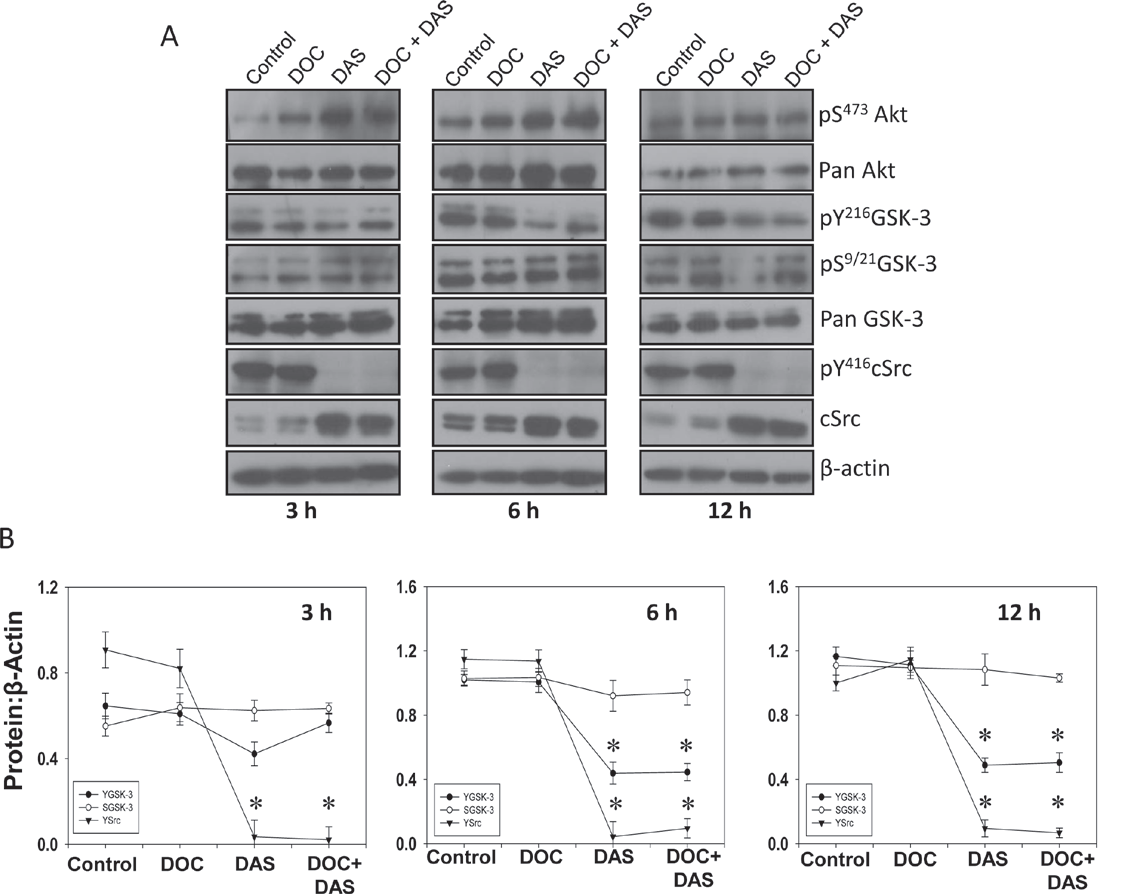
Dasatinib, a Src family kinases and Bcr/Abl inhibitor inhibits GSK-3 Y216 phosphorylation A) PC3 cells were treated with docetaxel (25 nM) and dasatinib (50 nM), respectively, or with a combination of docetaxel (25 nM) and dasatinib (50 nM) for 3 h, 6 h, and 12 h in serum free medium. Cell lysates from respective time points were subjected for Western blot analysis for changes in phosphorylation and expression levels of Akt, GSK-3 and Src. B) Bar graphs showing band densitometry analysis for phosphorylated and total Akt, GSK-3 and Src. The data are presented as mean ± SD (n=4) (* *p*<0.001, Δ *p*< 0.01, # *p*<0.05 vs. control experiments within the same group).

In order to confirm our findings on Src-mediated ^Y216^GSK-3 phosphorylation and its clinical relevance, we sought to determine the effect of pan SFK and Bcr/Abl inhibitor dasatinib in comparison to docetaxel which is approved by the US Food and Drug Administration (FDA) for hormone refractory prostate cancer. The results revealed that dasatinib, but not docetaxel, inhibits ^Y216^GSK-3 and ^Y416^Src phosphorylation in a time-dependent manner. Interestingly, this study also showed that dasatinib increases ^S473^Akt and ^S9/21^GSK-3 phosphorylation, but only at the first 3 h of treatment, whereas decreased phosphorylation of ^Y216^GSK-3 and ^Y416^Src starting from 6 h in a time-dependent manner (Figure 5A and B). Altogether, these results indicate that ^Y216^GSK-3 is a downstream target of activated Src tyrosine kinase.

### Src-^Y216^GSK-3 axis determines prostate cancer cells tumorigenic and metastatic functions in vitro and in vivo

**Figure 6:**
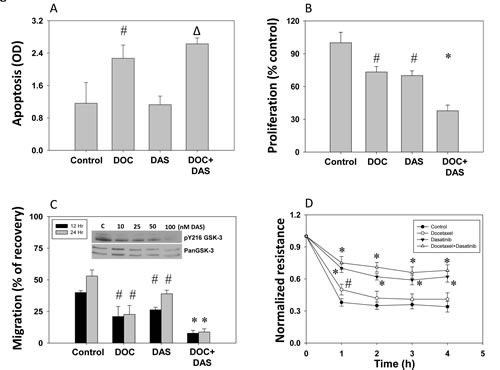
Dasatinib inhibits prostate cancer (PC3) cell apoptosis, proliferation, migration and micro-metastasis *in vitro* PC3 cells were treated with docetaxel (25 nM) and dasatinib (50 nM), or with a combination of docetaxel (25 nM) and dasatinib (50 nM) for 12 h in serum free medium. A) Bar graph showing increased apoptosis of PC3 cells after treatment with docetaxel, dasatinib and a combination of docetaxel with dasatinib determined based on cytoplasmic histone-associated DNA fragments detection. B) Bar graph showing decreased proliferation of PC3 cells after treatment with docetaxel, dasatinib and a combination of docetaxel with dasatinib as determined by the measurement of the amount of BrDU incorporation. C) Bar graph showing decreased migration of PC3 cells after treatment with docetaxel, dasatinib and a combination of docetaxel with dasatinib. Cells were grown to reach confluence and then serum starved for 3 h. A scratch assay for the assessment of motility was performed for 12 h and 24 h post treatment. Insert show Western blot images on the effect of various doses of dasatinib on pY^216^GSK-3 phosphorylation with no changes in total GSK-3 expression D) Trans-endothelial migration (micro-metastasis) of prostate cancer (PC3) cells measured using electric cell-substrate impedance sensing (ECIS) technology with human dermal micro-vascular endothelial cells plated on 8W10E array chips. Control PC3 cells and cells treated with docetaxel, dasatinib or a combination of docetaxel with dasatinib were collected from the plate by using cell dissociation buffer [20mM EDTA in PBS (pH=7.4)] were directly added onto the endothelial cell monolayer at a density of 5X10^4^ cells/well in 50 serum-free DMEM. Figure shows Real-Time measurements on the trans-endothelial migration of PC3 cells as recorded by the ECIS instrument up to 5 h. The data are presented as mean ± SD (n=4) (* *p*<0.001, Δ *p*< 0.01, # *p*<0.05 vs. control experiments within the same group).

To test the role of Src-^Y216^GSK-3 axis in prostate cancer, we first performed *in vitro* studies using PC3 cells treated with dasatinib alone or in combination with docetaxel as therapeutic agents and subjected those to different sets of experiments to establish their effects on tumorigenic and metastatic status. Our results showed that docetaxel, but not dasatinib, increased PC3 cell apoptosis. Although modest increase in PC3 cell apoptosis after dasatinib treatment was observed, the data were not statistically significant (Figure 5A). However, both docetaxel and dasatinib treatments resulted in decreased PC3 cells proliferation by about 30%, and, interestingly, an additive effect was noticed when docetaxel and dasatinib treatment was applied together (Figure 6B). Similarly to the results from proliferation assay, migration assay results revealed that both docetaxel and dasatinib significantly impair cell migration about 50%, with an additive effect when the drugs used in combination (Figure 6C). Finally, we determined the effect of dasatinib and docetaxel on micro-metastasis (transendothelial migration) of PC3 cells. Our experiment indicated that although both drugs exhibited the potential to inhibit PC3 cell motility and proliferation, only dasatinib treatment significantly inhibited micrometastasis (Figure 6D).

### Inhibition of Src-^Y216^GSK-3 axis by dasatinib inhibited growth of prostate tumor xenograft in athymic nude mice in vivo

**Figure 7:**
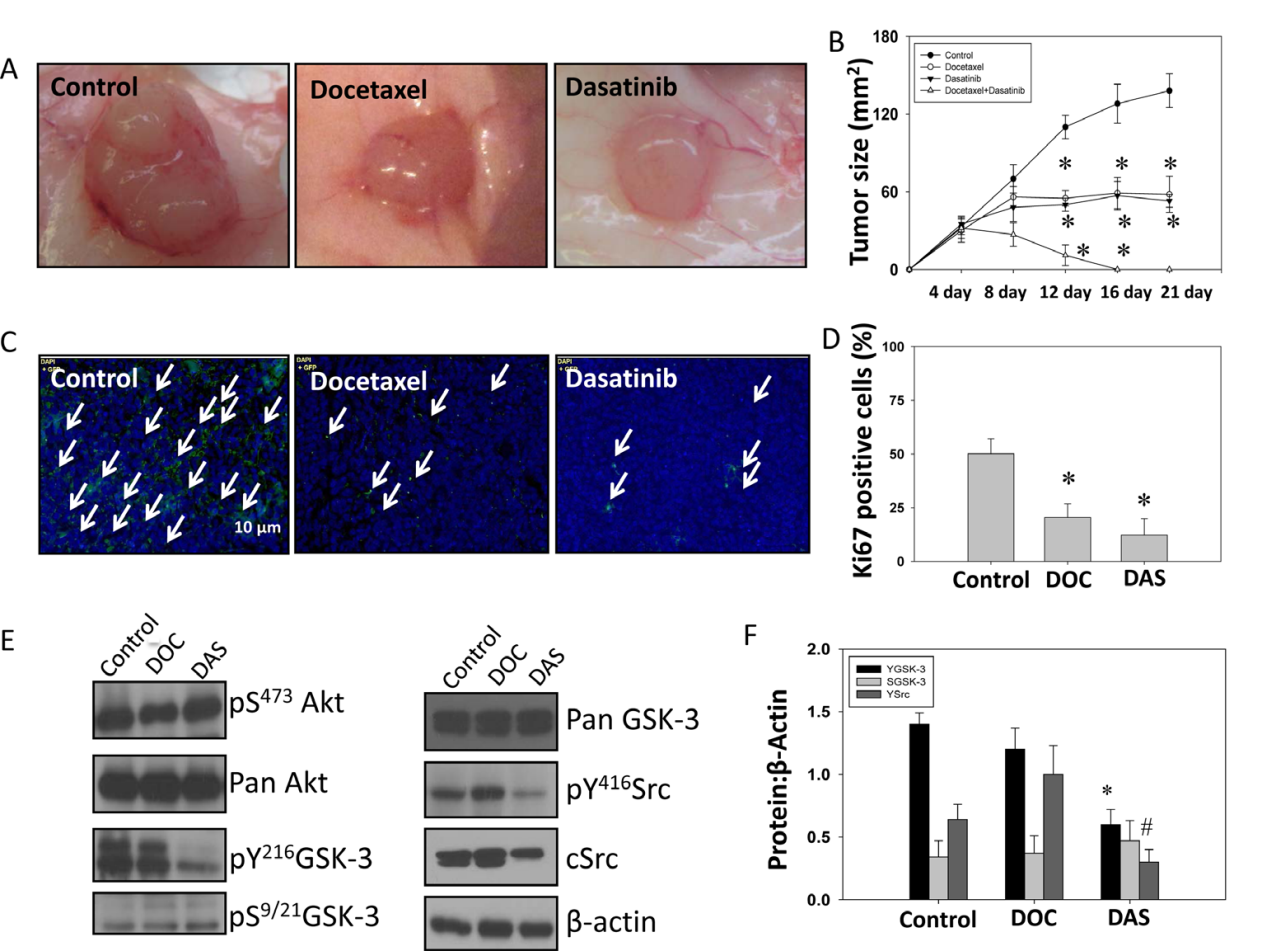
Dasatinib inhibits GSK-3 Y216 phosphorylation and growth of PC3 tumor xenograft in athymic nude mice *in vivo* PC3 cells were implanted subcutaneously into athymic nude mice at a concentration of 1.5 X 10^6^ cells in 100 μl of sterile saline. Mice were treated with drugs at the following doses; docetaxel (5 mg/kg), dasatinib (10 mg/kg) and a combination of docetaxel (5 mg/kg) with dasatinib (10 mg/kg). Docetaxel was administrated for 3 days per week for 2 weeks total, whereas dasatinib was administrated 5 days per week for 3 weeks total. A) Images showing tumor xenographs collected on day 21. B) Bar graph representing the tumor progression during drug administration for 21 days. The data are presented as mean ± SD (n=6). C) Images showing fluorescent immunohistochemistry of tumor xenograft frozen sections probed with Ki67 antibodies. D) Bar graph showing number of Ki67-positive cells on day 21 in tumor xenograft frozen sections from animals treated with sterile saline, docetaxel and dasatinib. The data are presented as mean ± SD (n=6). E) The western blot analysis of changes in phosphoryation and expression levels of Akt, GSK-3 and Src molecules in the tumor lysates. F) Bar graph showing densitometry analysis of the tumor lysates for Akt, GSK-3 and Src molecules. The data are presented as mean ± SD (n=4) (* *p*<0.001, Δ *p*< 0.01, # *p*<0.05 vs. control experiments within the same group).

To verify our findings from *in vitro* studies and test the efficacy of targeting Src-GSK-3 axis for prostate cancer therapy, we performed *in vivo* study using a PC3 cell tumor xenograft model in athymic nude mice followed by treatments with docetaxel and dasatinib, alone and in combination. Our data indicated that monotherapy with both docetaxel and dasatinib significantly inhibited prostate tumor growth *in vivo* by 50-70 % between days 12 and 21 (Fig. 7A and B). Moreover, our data indicated that treatment with either dasatinib or docetaxel resulted in significant inhibition of tumor cell proliferation as evidenced by the reduced Ki67 staining, with dasatinib showing superior inhibitory effects on PC3 cell proliferation compared to docetaxel (Figure 7C and D). To determine the involvement of Src-^Y216^GSK-3 axis in the regulation of prostate tumor growth, we subjected the frozen sections from control, docetaxel and dasatinib PC3 tumor xenografts to western blot analysis. Our analysis confirmed that levels of c-Src, ^Y416^Src, and ^Y216^GSK-3, but not ^S9/21^GSK-3 are reduced in dasatinib arm, but not in docetaxel arm (Figure 7E and F). Collectively, our study demonstrated the integral role of Src in mediating ^Y216^GSK-3 phosphorylation and subsequent activation leading for prostate cancer growth *in vivo*.

## DISCUSSION

Recent studies from our laboratory have demonstrated the integral role of Akt pathway in prostate cancer [3, 4, 6]. Although Akt is known to phosphorylate a plethora of substrates, exact functions of Akt and its various known and yet to be identified substrates are currently under investigation. Two-gene family of glycogen synthase kinase-3 (GSK-3α and GSK-3β) is one of the less investigated substrates of Akt for its role in physiology and pathology. Due to the indispensable role of Akt in cancer and activity inhibiting GSK-3 phosphorylation by Akt at its Ser21/9 residue, suppression of GSK-3 activity was assumed to be tumor promoting. However, recent studies have revealed the tumor promoting effects of GSK-3 activation in multiple cancers [12, 26, 38-40]. This suggested the existence of an alternate mechanism that can sustain GSK-3 activity in cancer cells despite the inhibitory phosphorylation by Akt. Here we demonstrate the importance of Src-mediated phosphorylation of ^Y216^GSK-3 in its activity regulation, and in mediating multiple prostate cancer cellular function *in vitro* and tumor growth *in vivo*. Our data indicated that inhibition of GSK-3 activity as a result of treatment with GSK-3 specific inhibitor SB415286 or SiRNA-mediated knockdown of GSK-3 in murine TRAMP (TR-C2N) and human (PC3) invasive prostate cancer cell lines lead to impaired prostate cancer cell motility, proliferation, survival, invasion and colony formation *in vitro*. While expression of PC3 cells with constitutively active cSrc (CA-Src) resulted in enhanced ^Y216^GSK-3 phosphorylation, reduced ^Y216^GSK-3 phosphorylation was observed by expression with kinase dead Src (DN-Src). Analysis of SYF mouse embryonic fibroblasts that are deficient in cSrc, Yes and Fyn showed that even in normal cells Src deficiency is correlated with reduced ^Y216^GSK-3 phosphorylation. Reconstituting Src activity by expression with CA-Src in SYF cells significantly restored ^Y216^GSK-3 phosphorylation, once again indicating that ^Y216^GSK-3 is a target of Src activity. Treatment of PC3 cells with dasatinib,an inhibitor of SFKs and Bcr/Abl, but not with docetaxel, resulted in reduced phosphorylation of ^Y416^Src and ^Y216^GSK-3. Even though treatment with dasatinib did not significantly induce apoptosis in PC3 cells, this treatment resulted in significant impairment in cell motility, proliferation and transendothelial migration (micrometastasis). Furthermore, treatment with dasatinib resulted in decreased ^Y416^Src and ^Y216^GSK-3 phosphorylation, reduced rate of cell proliferation and growth of PC3 tumor xenograft in an athymic nude mouse model. Together, we demonstrate a novel mechanism of GSK-3 activity regulation in normal and cancer cells with its specific role in prostate cancer progression.

Historically, changes in GSK-3 activity has been investigated based on the changes in phosphorylation of Ser21 and Ser9 of GSK-3α and GSK-3β, respectively by Akt [10] and to a lesser extent by a few other kinases such as casein kinase-2 [41], protein kinase [42] and protein kinase C [43]. While p38 MAP kinase has been indicated in the negative regulation of GSK-3β activity via phosphorylation of Ser389 and Thr390 [44], ERK has been shown to inhibit GSK-3β activity by phosphorylation of Thr43 [45]. Conversely, GSK-3 is activated by phosphorylation of Tyr216 in GSK-3*β* and Tyr-279 in GSK-3*α present in its “T-Loop”* found within subdomain VIII [13-15]. But the mechanism regulating GSK-3 tyrosine phosphorylation is not yet fully characterized and appears to be cell type and context dependent. While a number of candidates such as ZAK1 [46], Fyn [47], and Pyk2 [48, 49] have been reported to be responsible for ^Y216^GSK-3 phosphorylation in various cell types, scientists also debate the possibility of GSK-3β un-phosphorylated at its Ser9 can act as a tyrosine kinase, auto-phosphorylate its Y216 residue and then transform into a serine-threonine kinase [14, 50].

Crystal structure study has shown that ^Y216^GSK-3β phosphorylation induces conformational changes in the kinase enhancing its interaction with the substrate, and GSK-3β un-phosphorylated at Y216 does not interact with its substrate [51]. Many studies indicate that over-expression of cells with plasmids encoding GSK-3β S9A mutant, which is currently considered as constitutively active GSK-3, leads to hyper-activation of the GSK-3 signaling pathway [52, 53]. Hence, in a scenario where GSK-3 Y216 phosphorylation has to be considered as an autophosphorylated event, it appears that dephosphorylation of its Ser9 is a pre-requisite. While autophosphorylation by GSK-3 at its Y216 residue cannot be ruled out in certain conditions, at least in cancer cells, where kinases such as Akt and PKC are hyper-activated, sustaining GSK-3 activity can happen only with the existence of an alternate pathway, potentially involving a non-receptor tyrosine kinase.

Although deregulation of PI3 Kinase-Akt pathway as a result of PTEN mutation is one of the major cause for prostate cancer [1, 54, 55], enhanced expression and activity as well as changes in intracellular localization of both GSK-3α and GSK-3β have also been correlated with the Gleason score of the prostate cancer patients [23], once again suggesting that an Akt-independent pathway does exist in prostate cancer cells in the regulation of GSK-3 activity. In addition to Akt and GSK-3, Src is an additional kinase that has been implicated in prostate cancer [56-58]. Our data indicate that modulation of Src activity using constitutively active and kinase-dead Src plasmids in prostate cancer cells results in the modulation of ^Y216^GSK-3β phosphorylation. Reduced phosphorylation of ^Y216^GSK-3β phosphorylation was also observed in SYF fibroblasts indicating that even in fibroblasts, where Src is essential for wound healing and modulation of fibrotic events [59], GSK-3 activity can be modulated by Src kinases. Furthermore, treatment of prostate cancer cells with dasatinib, a Src and Bcr-Abl inhibitor, but not with docetaxel, resulted in reduced ^Y216^GSK-3β phosphorylation as well as impaired cellular function *in vitro* and tumor growth *in vivo*. Together, our study for the first time, demonstrated the existence of a Src-mediated ^Y216^GSK-3β phosphorylation and activation leading to prostate cancer cell motility, proliferation, micrometastasis and tumor progression. Most importantly, information obtained from our study could be useful in the development of novel therapeutic strategies for the treatment of early and castration resistant prostate cancer.

## MATERIAL AND METHODS

### Reagents, cell lines and antibodies

*Murine* TRAMP (TR-C2N) cell line (gifted by Dr. Warren Heston, Cleveland Clinic), h*uman PC3 cells (ATCC, Manassas, VA) and* “SYF” cells (mouse embryonic fibroblasts deficient in Src, Yes and Fyn) were maintained in DMEM (HyClone, Thermo Scientific, Logan, UT) with 10% fetal bovine serum, 100 units/ml penicillin, and 100 μg/ml streptomycin in a 5% CO_2_ atmosphere at 37°C. Primary antibodies against phospho-Akt^Ser473^, phospho-GSK-3α/β^Ser9/21^, phospho-Src^Tyr416^, phospho-βcatenin^Ser33/37/Thr41^, pan-Akt and siRNA for GSK-3α/β were purchased from Cell Signaling (Boston, MA). Phospho-GSK-3^Tyr216^ was obtained from BD Biosciences (Franklin Lakes, NJ). Primary antibodies against β-actin and GSK-3 inhibitor (SB415286) were purchased from Sigma, St. Louis, MO. All secondary antibodies were obtained from Bio-Rad (Hercules, CA). EGF was obtained from R&D (Minneapolis, MN). Docetaxel was purchased from Sigma, St. Louis, MO and Dasatinib was purchased from Santacruz biotechnology, (Dallas, TX).

### Transfections

Human PC3 cells were transiently transfected with either SiRNA-GSK-3 (Santacruz biotechnology, Dallas, TX) using oligofectamine (Life technologies, Carlsbad, CA) or cSrc constructs for CA-Src (Y527F) and DN-Src (K259R), and Akt constructs for CA-Akt (myr-Akt) and DN-Akt (Akt-K179M) using lipofectamine 2000 (Life technologies, Carlsbad, CA) as transfection reagents according to the manufacturer's protocol. SYF cells were transiently transfected with CA-Src construct using lipofectamine 2000. Control cells were transfected with either scrambled SiRNA or empty vector pBabe-puromycin. Approximately 75–85% transfection efficiency was obtained.

### *In vitro* apoptosis assay

Apoptosis assay was performed as previously described [5] and determined based on cytoplasmic histone-associated DNA fragments detection, quantified by the Cell Death Detection ELISA^PLUS^ kit (Roche Applied Science, Indianapolis, IN) according to the manufacturer's protocol. Briefly, TRAMP (TR-C2N) and PC3 cells were plated in 96-well plate at a density of 10^4^ cells/well. After 24h, TRAMP cells were treated with 50 nM EGF plus 20 μM of SB415286 for 12 and 24 h. Control cells received only 50 nM EGF. PC3 cells were transiently transfected with SiGSK-3 and scrambled SiRNA (control), or received treatment as follows: docetaxel (25 nM) and dasatinib (50 nM) or a combination of docetaxel (25 nM) with dasatinib (50 nM). Next, cells were lysed and centrifuged at 200*g* for 10 min, and the collected supernatant was subjected to apoptosis detection ELISA plate. The absorbance was measured at 405 nm (reference wavelength, 492 nm). The data are presented as mean ± SD (n=4).

### *In vitro* and *In vivo* proliferation assay

Proliferation assay was carried out *in vitro* and *in vivo* as previously described [5]. Briefly, proliferation assay *in vitro* was determined using the nonradioactive BrDU-based cell proliferation assay (Roche, Basel, Switzerland) according to the manufacturer's protocol. Briefly, TRAMP (TR-C2N) and PC3 cells were seeded in 96-well plates at a density of 5x10^3^cells per well. After 24 h, TRAMP cells were treated with 50 nM EGF plus 20 μM of SB415286 for 12 and 24 h. Control cells received only 50 nM EGF. PC3 cells were transiently transfected with SiGSK-3 and scrambled SiRNA (control), respectively, or received followed treatment: docetaxel (25 nM) and dasatinib (50 nM) or a combination of docetaxel (25 nM) with dasatinib (50 nM). Next, cells were subjected to a 5-bromo-2-deoxyuridine assay using the BrDU Labeling and Detection Kit III (RocheApplied Science), according to the manufacturer's protocol. BrDU incorporation into the DNA was determined by measuring the absorbance at both 450 and 690 nm on ELISA plate reader. Proliferation assay *in vivo* was performed using immunofluorescence staining of the tumor sections for Ki67 antigen as a marker of proliferating cells performed according to the protocol of the manufacturer (Sigma, St. Louis, MO). Briefly, formalin-fixed, frozen prostate (PC3) xenograft tumor sections from nude mice were subjected to the standard xyline ethanol dehydration process and permeabilized with 0.3% Triton X-100 in 1 X PBS. The nonspecific staining was blocked with 5% goat serum for 1 h at room temperature. The dehydrated, permeabilized, and blocked tissue sections were incubated with primary antibody against Ki67 antigen (dilution 1:1000) overnight at 4 °C, followed by washing with 1 X PBS (4 times for 15 min each). Next, anti-mouse Alexa Fluor 488-labeled secondary antibodies (1:500) were applied for 1 h at room temperature and washed four times for 15 min with 1 X PBS. The slides were mounted with Vectashield (Vector Laboratories), and thei mages were taken by a Zeiss fluorescent microscope (ZeissAxiovert100M, Carl Zeiss). The data are presented as mean ± SD (n=4).

### Colony formation assay

Colony formation assay was performed using standardized protocol in the lab [3]. In this approach, TRAMP cells (TR-C2N) and PC3 cells were cultured on 6-well plates. After 24 h, TRAMP cells were treated with 50 nM EGF plus 20 μM of SB415286 for 16 h. Control cells received only 50 nM EGF. PC3 cells were transient transfected with siRNA-GSK-3 whereas control cell were transient transfected with scrambled siRNA. 5 days later, each of the wells was fixed using methanol and stained by crystal violet. Next, cells were counted using Image J software for the number of colonies which were compared to the control. The data are presented as mean ± SD (n=3).

### Migration assay

Migration assay was performed as previously described [6]. Briefly, TRAMP cells (TR-C2N) were plated in 12-well plates and after reaching monolayer treated with either 50 nM EGF plus 20 μM of SB415286. Control cells received only 50 nM EGF. PC3 cells were transient transiently transfected with SiGSK-3 and scrambled SiRNA (control) or received followed treatment: docetaxel (25 nM), dasatinib (50 nM) or a combination of docetaxel (25 nM) with dasatinib (50 nM). Next, cells were grown on plates to reach confluence (approximately 16 h). A scratch was made in the monolayer and pictures were taken at 0, 12 h and 24 h. The rate of migration (as measured by scratch recovery) was calculated using the following equation (1-T_t_/T_0_) X 100, were t = 12 h or 24 h. The data are presented as mean ± SD (n=4).

### Invasion assay

The invasion assay was performed as previously described [4] using BD BioCoat Tumor Invasion Kit (BD Biosciences) coated with BD Matrigel Matrix according to the manufacturer's protocol. Briefly, TRAMP (TR-C2N) and PC3 cells transiently transfected with either scrambled SiRNA or SiGSK-3 were labeled for 30 min with BD DilC12 fluorescence dye and seeded onto the upper chamber of 96-well Transwell plate at a density of 1x10^4^ cells per well in 400 μl serum free medium. Serum free medium was then added to the lower chamber of TRAMP cells and supplemented with 50 nM EGF plus 20 μM of SB415286. Control cells were treated with only 50 nM EGF. Serum free medium added to the lower chamber of PC3 cells as well and then supplemented only with 50 nM EGF. The absorbance of the stained cells was measured after 12 and 24 h on an ELISA plate reader. The data are presented as mean ± SD (n=4).

### Micro-metastasis assay

Trans-endothelial migration of prostate cancer (TRAMP and PC3) cell lines was measured using Electric Cell-substrate Impedance Sensing (ECIS) equipment with human dermal microvascular endothelial (TIME) cells (ATCC, *Manassas, VA*) plated on 8W10E+ array chips (Applied Biophysics, Troy, NY) as previously described [6]. Following this, PC3 were treated with docetaxel (25 nM), dasatinib (50 nM) or a combination of docetaxel (25 nM) with dasatinib (50 nM). Next, cells were directly added onto endothelial cell monolayer at a density of 5x10^4^ cells per well in 50 μl medium. Cells were detached from plate by using cell dissociation buffer (20 mM EDTA in PBS, pH 7.4) to avoid integrin/receptor loss due to trypsin digestion. Real-time measurements on the trans-endothelial migration of PC3 cells were recorded by the ECIS instrument up to 12 h and changes in the initial 5 h was analyzed.

### Drug formulation and *in vivo* administration

Docetaxel and dasatinb were dissolved in 80 mmol/L citrate buffer (pH=3.1) according to the manufacture's instruction to a concentration of 20 mg/ml. For injections, PC3 cells were grown to confluence in 250-ml flasks. Next, cells were re-suspended in sterile saline to a concentration of 2.0 106/ml. Cell suspension (100 μl) was injected subcutaneously into 8-week-old nude mice (athymic nude mice; Harlan, Indianapolis, IN). Four days after xenograft injection, mice were randomized to receive drug or control vehicle (6 mice per group). The controls animals were injected intra-peritoneally with 100 μl of sterile saline, whereas treatment animals received followed equal volume treatment of docetaxel (5 mg/kg) and dasatinib (10 mg/kg), respectively, or in combination of docetaxel (5 mg/kg) and dasatinib (10 mg/kg). Docetaxel was administrated for 3 days per week for 2 weeks total, whereas dasatinib was administrated 5 days per week for 3 weeks total. Mice were sacrificed on day 21 and tumors were dissected, weighed, and snap-frozen using dry ice for further processing to use in Western blot analysis and immunohistochemistry. All animal procedures were performed according to the protocol approved by the Institutional Animal Care and Use Committee.

### Western blot analysis

Whole cell lysates were prepared using lysis buffer [50 mM Tris-HCl (pH=7.4), 1% TritonX-100, 150mM NaCl, 1mM EDTA, 2mM Na_3_VO_4_, and 1X Complete protease inhibitors (Roche Applied Science, Indianapolis, IN)]. The protein concentration was measured by the D_c_ protein assay (Bio-Rad, Hercules, CA). Western analyses were performed using standard Laemmle's method as done previously [3]. Densitometry was done using NIH Image J software.

### Statistical Analysis

All the data are presented as means ± SD (n = 3 to 6). To determine significant differences between treatment and control values, we used the Student's two-tailed *t* test. The significance was set at 0.05 levels (marked with symbols wherever data are statistically significant).

## Supplementary Figure


