# Supplementary material for: Targeting Src-mediated Tyr216 phosphorylation and activation of GSK-3 in prostate cancer cells inhibit prostate cancer progression in vitro and in vivo
Source: Oncotarget. 2014 Feb 7;5(3):775–87. doi: 10.18632/oncotarget.1770 (PMC3996673; doi:10.18632/oncotarget.1770)
Supplement: Supplementary file 1 [file oncotarget-05-775-s001.pdf]

Targeting Src-mediated Tyr216 phosphorylation and activation of GSK-3 in prostate cancer cells inhibit prostate cancer progression *in vitro* and *in vivo* – *Goc et al*

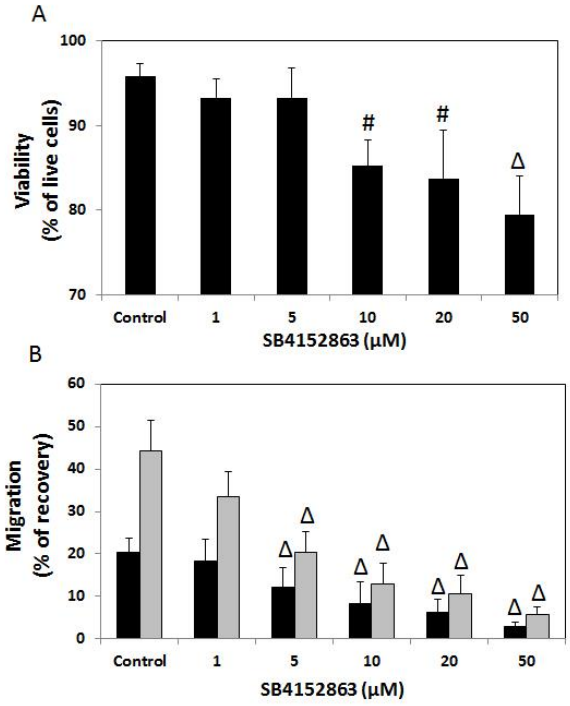

Supplemental Figure 1: GSK-3 inhibitor SB4152856 inhibits PC3 cell viability and migration in a dose-dependent manner. PC3 cells were treated with various doses of SB415286 (0, 1, 5, 10, 20 and 50 µM) and subjected for Trypan blue staining to determine cell viability at 12 h after treatment. A) Bar graph showing decreased cell viability PC3 cells after treatment with GSK-3 inhibitor with a peak inhibition observed at 20 µM of SB415286. B) Bar graph showing decreased motility of PC3 cells after treatment with various doses of GSK-3 inhibitor SB415286 (0, 1, 5, 10, 20 and 50 µM) with a peak inhibition observed at 20 µM . The data are presented as mean ± SD (n=4) of triplicate experiments (Δ $p< 0.01$, # $p<0.05$ vs. control experiments within the same group).
